# Proteomic Analysis of Non-human Primate Peripheral Blood Mononuclear Cells During *Burkholderia mallei* Infection Reveals a Role of Ezrin in Glanders Pathogenesis

**DOI:** 10.3389/fmicb.2021.625211

**Published:** 2021-04-22

**Authors:** Chih-Yuan Chiang, Yang Zhong, Michael D. Ward, Douglas J. Lane, Tara Kenny, Raysa Rosario-Acevedo, Brett P. Eaton, Sylvia R. Treviño, Taylor B. Chance, Meghan Hu, Patricia L. Worsham, David M. Waag, Richard T. Moore, Lisa H. Cazares, Christopher K. Cote, Yingyao Zhou, Rekha G. Panchal

**Affiliations:** ^1^Countermeasures Division, United States Army Medical Research Institute of Infectious Diseases, Frederick, MD, United States; ^2^Genomics Institute of the Novartis Research Foundation, San Diego, CA, United States; ^3^Systems and Structural Biology Division, Protein Sciences Branch, United States Army Medical Research Institute of Infectious Diseases, Frederick, MD, United States; ^4^Bacteriology Division, United States Army Medical Research Institute of Infectious Diseases, Frederick, MD, United States; ^5^Pathology Division, United States Army Medical Research Institute of Infectious Diseases, Frederick, MD, United States

**Keywords:** *Burkholderia mallei*, glanders, innate immunity, inflammatory responses, biothreat agent, proteomics

## Abstract

*Burkholderia mallei*, the causative agent of glanders, is a gram-negative intracellular bacterium. Depending on different routes of infection, the disease is manifested by pneumonia, septicemia, and chronic infections of the skin. *B. mallei* poses a serious biological threat due to its ability to infect via aerosol route, resistance to multiple antibiotics and to date there are no US Food and Drug Administration (FDA) approved vaccines available. Induction of innate immunity, inflammatory cytokines and chemokines following *B. mallei* infection, have been observed in *in vitro* and small rodent models; however, a global characterization of host responses has never been systematically investigated using a non-human primate (NHP) model. Here, using a liquid chromatography-tandem mass spectrometry (LC-MS/MS) approach, we identified alterations in expression levels of host proteins in peripheral blood mononuclear cells (PBMCs) originating from naïve rhesus macaques (*Macaca mulatta*), African green monkeys (*Chlorocebus sabaeus*), and cynomolgus macaques (*Macaca fascicularis*) exposed to aerosolized *B. mallei*. Gene ontology (GO) analysis identified several statistically significant overrepresented biological annotations including complement and coagulation cascade, nucleoside metabolic process, vesicle-mediated transport, intracellular signal transduction and cytoskeletal protein binding. By integrating an LC-MS/MS derived proteomics dataset with a previously published *B. mallei* host-pathogen interaction dataset, a statistically significant predictive protein-protein interaction (PPI) network was constructed. Pharmacological perturbation of one component of the PPI network, specifically ezrin, reduced *B. mallei* mediated interleukin-1β (IL-1β). On the contrary, the expression of IL-1β receptor antagonist (IL-1Ra) was upregulated upon pretreatment with the ezrin inhibitor. Taken together, inflammasome activation as demonstrated by IL-1β production and the homeostasis of inflammatory response is critical during the pathogenesis of glanders. Furthermore, the topology of the network reflects the underlying molecular mechanism of *B. mallei* infections in the NHP model.

## Introduction

*Burkholderia mallei*, a gram-negative intracellular bacterium, is the etiological agent of glanders. Equids are natural reservoirs of *B. mallei* and clinical signs of glanders in equids include chronic nasal discharge, enlargement of the lymph node and the presence of nodules, pustules, or ulcers on the flanks and extremities of infected animals (Galyov et al., 2010). Personnel who work in close proximity with infected animals or manipulate the organism in laboratory settings are at risk of exposure. Treatment of glanders usually involves extended regimens of multiple antibiotics (Lipsitz et al., 2012). However, differences in antibiotic susceptibility patterns in *B. mallei* isolates may complicate the design of effective regimens and relapse can occur. Given that *B. mallei* is resistant to multiple antibiotics, elicits both acute and chronic infection and could potentially be used as a bioweapon, it is categorized a Tier 1 select biological agent by both the US Department of Health and Human Services and the US Department of Agriculture. Development of countermeasures against this biothreat agent will require FDA approval under the animal rule in lieu of clinical trials. Hence, better understanding of the disease progression and host-pathogen interactions using proteomic analysis, in a well-characterized animal model such as Non-human primates (NHPs), which closely mimic human infection, is critical.

*B. mallei* enters the phagosome through phagocytosis. Subsequently, bacteria escape from vacuolar membranes and enter into the host cytosol. Once free, bacteria exploit host factors (HFs) to trigger actin polymerization and induce multinucleated giant cells (MNGCs) (Galyov et al., 2010). Following entry into adjacent cells, bacteria escape from secondary phagosomes, replicate and spread. Engagement of *B. mallei* with pattern recognition receptors (PRRs) induces the expression of cellular protective reactive nitrogen oxide species (RNOS), multiple cytokines and chemokines (Chiang et al., 2015). Inducible nitric oxide synthase (iNOS) mediates the production of high levels of nitric oxide (NO), which is subsequently oxidized to bactericidal RNOS. iNOS inhibitor treatment significantly impairs the clearance of *B. mallei* infection and iNOS deficient mice succumb to *B. mallei* infection 30 days post-infection (Brett et al., 2008; Rowland et al., 2010). Both type I (IFN-β) and type II (IFN-γ) interferons (IFNs) are also reported to modulate *B. mallei* infection. In RAW264.7 macrophages, *B. mallei* clearance correlates with both IFN-β production and iNOS activity (Brett et al., 2008). The role of IFN-γ was demonstrated in an *in vivo* mouse model where IFN-γ knockout mice were more susceptible to *B. mallei* infection. Similarly, monocyte chemoattractant protein-1 (MCP-1) and MCP-1 receptor (CCR2) deficient mice were defective in up-regulating IFN-γ, which may ultimately contribute to their inability to clear *B. mallei* infection (Goodyear et al., 2010). In addition to MCP-1, interleukin (IL)-12 (IL-12) and IL-18 were also involved in regulating IFN-γ production in response to heat-killed *B. mallei* (Rowland et al., 2006). Importantly, upon *B. mallei* infection, mice that are deficient of MyD88, an adaptor molecule that mediates TLR signaling activation, failed to produce IFN-γ, which may lead to elevated bacterial load and significantly short survival times (Goodyear et al., 2012). Taken together, a functional innate immune system is critical for the efficient, early control of *Burkholderia* infection and key cytokines including MCP-1, INF-β, and INF-γ are required for protective immunity to *Burkholderia* infection.

Infection with *B. mallei* in multiple animals species including mice (*mus musculus*), Syrian hamsters (*Mesocricetus auratus*), and marmoset (*Callithrix jacchus*) showed clinical symptoms that resemble human glanders (Fritz et al., 1999; Lever et al., 2003; Lafontaine et al., 2013; Jelesijevic et al., 2015). Typical symptoms include splenic, hepatic, and nasal lesions (Lever et al., 2003; Jelesijevic et al., 2015), peritonitis, splenomegaly, pyogranulomatous inflammation of the spleen and lung and subcutaneous hemorrhage/edema (Fritz et al., 1999; Jelesijevic et al., 2015). Although animal model studies provide insight toward the pathogenesis of *B. mallei* infection, the underlying molecular mechanism that governs the infection has not been elucidated. Previous efforts toward global understanding of host—*B. mallei* infection were based on the yeast two-hybrid (Y2H) assay to generate a comprehensive protein-protein interaction (PPI) map between each of the 49 *B. mallei* virulence factors (VFs) and either human or mouse proteins (Memišević et al., 2013).

The goal of this research is to utilize a LC-MS/MS approach to profile the changes in protein expression in PBMCs derived from *B. mallei* infected and uninfected NHPs (rhesus macaques, African green monkeys and cynomolgus macaques) and gain insight toward the underlying molecular network that governs *B. mallei* pathogenesis. This methodology has identified critical signaling components and molecular pathways that modulate pathogen infections in NHPs (Baas et al., 2006; Gravett et al., 2007; Brown et al., 2010). Notably, ezrin was identified as a critical signaling component in modulating *B. mallei* infection in the *in vitro* model. The ezrin inhibitor specifically impinged on *B. mallei* mediated IL-1β and IL-1Ra expression levels. Taken together, proteins that were differentially expressed after *B. mallei* infection provided a comprehensive view between cellular host proteins and *B*. *mallei* VFs.

## Materials and Methods

### Reagents and Cell Culture

Ezrin inhibitor (NSC668394) was purchased from EMD Millipore (Bulut et al., 2012). V-PLEX Custom Mouse Cytokine immunoassay kits were purchased from Meso Scale Discovery. RAW264.7 macrophages (American Type Culture Collection) were maintained at 37°C, 5% CO_2_ in growth medium: Dulbecco’s Modified Eagle Medium (Thermo Fisher Scientific, CA) supplemented with 10% (v/v) fetal bovine serum (HyClone, UT) and 1% penicillin and streptomycin (Thermo Fisher Scientific, CA).

### Strain Selection

*B. mallei* FMH (China 7) was selected for the glanders study. It is a human isolate obtained from a patient with a laboratory-acquired infection. This strain was chosen based on association with human disease, virulence in animal models, availability of genetic analyses, and passage history (Waag et al., 2012). *B. mallei* used for aerosol exposure was grown in glycerol tryptone broth (1% tryptone, 0.5% NaCl, 4% glycerol) at 37°C with shaking at 200 rpm for approximately 18 h.

### Animal Study

**FIGURE 1:**
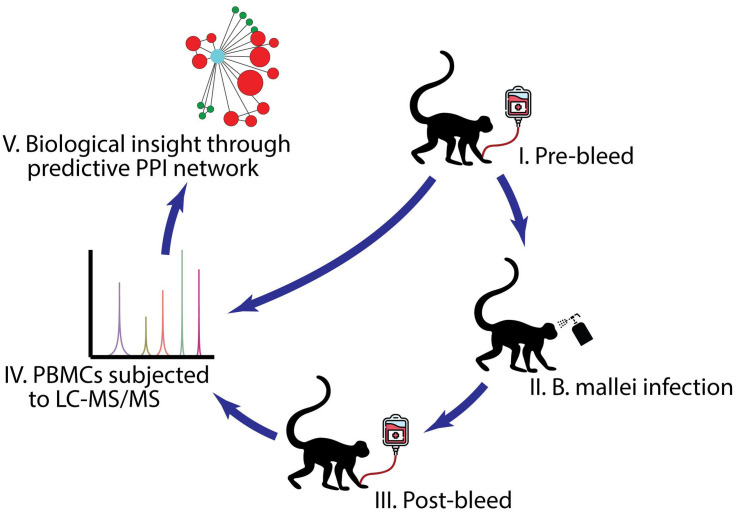
A schematic diagram of the experimental design. (I) PBMC samples were collected from rhesus macaques (*n* = 6), African green monkeys (*n* = 4), and cynomolgus macaques (*n* = 5) before *B. mallei* infection. (II) NHPs were infected with *B. mallei* at a CFU = 2.3 × 10^7^ via the aerosol route. (III) PBMC samples were collected from rhesus macaques, African green monkeys and cynomolgus macaques either at the time of euthanasia based on clinical score or those that survived till the end of study design. (IV) PBMCs purified from the surviving NHPs were subjected for LC-MS/MS analyses and the abundance of proteins within biological samples were identified. (V) A host-*B. mallei* virulence factor protein-protein interaction network was constructed. Icons- Monkey, spray bottle and transfusion bag were made by Freepik. https://www.flaticon.com/authors/freepik.

Six animals from each of the three NHP species (rhesus macaques, African green monkeys, cynomolgus macaques) were included in the study. Experimentally naïve NHPs were screened before being placed on protocol (e.g., chest radiograph, clinical chemistry and hematology, and a physical exam) to ensure the general health status of the animals is considered normal and that no underlying infection or abnormal health conditions are present that could adversely affect the study or data interpretation. The NHPs were then randomly assigned and balanced with regards to sex to obtain an approximate equal ratio of males and females in each experimental cohort. Under biosafety level 3 (BSL-3) conditions, the NHPs were exposed to a challenge dose of approximately 2.3 × 10^7^ aerosolized colony forming units (CFU) of *B. mallei* FMH23344 (dose range 1.51 × 10^7^ to 3.43 × 10^7^). Briefly, prior to aerosol exposure, NHPs were anesthetized by intramuscular injection with a ketamine/acepromazine mixture (10:1) for African green monkeys or Telazol was used to anesthetize the macaque species. Minute volumes (MV) were estimated using plethysmography. NHPs were exposed to a small-particle aerosol in a well-characterized, dynamic, head-only exposure chamber controlled and monitored by an automated bioaerosol exposure system. Integrated air samples were obtained from each run using all-glass impingers. Individual aerosol exposure doses were calculated as the product of the average aerosol concentration within the chamber and the total volume of air breathed by the NHP (Figure 1).

### Peripheral Blood Mononuclear Cell Isolation From Blood

Blood samples were collected from rhesus macaques (*n* = 6), African green monkeys (*n* = 4), and cynomolgus macaques (*n* = 5) before infection and then post infection either at the time of euthanasia based on clinical score or those that survived till the end of study design (42–45 days post-exposure) (Supplementary Table 1). Buprenorphine was administered to all animals post-exposure when clinical score deemed necessary in an attempt to alleviate pain and/or distress associated with the infection. The blood was collected in sodium heparin without EDTA and centrifuged for 15 min at 2,400 rpm. The cell pellet was subjected to density gradient separation on Ficoll Paque (GE Healthcare Life Sciences, Buckinghamshire, United Kingdom) and centrifuged at 2,270 rpm for 25 min at room temperature. After centrifugation, the PBMC layer was collected, washed in Hank’s Balanced Salt Solution (HBSS) and then stored at −80°C for subsequent proteomic studies.

### Liquid Chromatography-Tandem Mass Spectrometry

Briefly, 1 × 10^6^ PBMCs were collected and lysed in T-per reagent (Thermo Fisher Scientific, CA) containing protease inhibitors. After a brief centrifugation step to remove cellular debris, SDS-PAGE sample buffer (Thermo Fisher Scientific, CA, 4X) containing DTT was added to the cleared lysate. Samples were heated to 95°C for 15 min to inactivate the bacteria. Each PBMCs protein lysate was gel-fractionated by running on a 4–12% Tris-Bis polyacrylamide gel and dividing the gel lane into 10 equal fractions. In-gel trypsin digestion was then performed and the resulting peptides were analyzed by LC-MS/MS on a LTQ Orbitrap Elite mass spectrometer equipped with a Dionex 3000 RSLCnano system (Thermo Fisher Scientific). The system injected 5 μL of each digest onto a pre-column (C18 PepMap 100, 5 μm particle size, 5 mm length × 0.3 mm internal diameter) housed in a 10-port nano switching valve using a flow rate of 10 μl/min. The loading solvent was 0.1% formic acid in HPLC grade water. Peptides were then loaded onto an Easy-Spray analytical column (15 cm × 75 μm) packed with PepMap C18, 3 um particle size, 100A porosity particles (Thermo Fisher Scientific, CA). A 2–58% B gradient elution in 60 min was formed using Pump-A (0.1% formic acid) and pump-B (85% acetonitrile in 0.1% formic acid) at a flow rate of 300 nL/min. The column eluent was connected to an Easy-Spray source (Thermo Fisher Scientific, CA) with an electrospray ionization voltage of 2.2 kV. An Orbitrap Elite mass spectrometer (Thermo Fisher Scientific, CA) with an ion transfer tube temperature of 300°C and an S-lens setting of 55% was used to focus the peptides. A top 10 data dependent MS/MS method was used to select the top 10 most abundant ions in a 400–1,600 amu survey scan (60,000 resolution FWHM at m/z 400) with a full AGC target value of 1e6 ions and a maximum injection time of 200 ms. Higher Energy Collisional Dissociation (HCD) ms/ms spectra were acquired at a resolution of 30,000 (FWHM at m/z 400) with an AGC target value of 5e4 ions and a maximum injection time of 200 ms. MS^2^ data from all 10 fractions were then merged for the protein database search, performed with MASCOT^TM^ 1.4 (Matrix Science, London GB), using a mixed taxonomy (NHP and *Burkholderia*) subset database of the non-redundant protein database from National Center for Biotechnology Information (NCBI) web site^1^. Search parameters included trypsin enzyme specificity with two missed cleavages, variable modifications included oxidized methionine, precursor mass tolerance of 20 ppm and product ion mass tolerance of 0.5 Da. Only proteins with a minimum of 2 unique peptides were included in the final dataset. Expression levels were based on the Exponentially Modified Protein Abundance Index (emPAI) which is available in the MASCOT software package. The emPAI score offers approximate, label-free, relative quantitation of the proteins in a mixture based on protein coverage by the peptide matches in a database search result. The mass spectrometry proteomics data has been deposited in the ProteomeXchange Consortium via the PRIDE partner repository with the dataset identifiers listed in Supplementary Table 6.

### Identification of Differentially Expressed Proteins

Prioritization process of 3,288 human genes: Genes with neither pre- nor post-infection emPAI for one host were considered absent and not used for the fold change (FC) calculation of the same host; however, they might be retained for other hosts. A noise level of 0.03 was determined by the 5th percentile of all data and any emPAI less than the noise level was set to 0.03 for the following analysis. Fold changes of emPAIs between pre- and post-infection were calculated for remaining genes in 15 donors of three species. *P-*value based on Redundant siRNA Analysis (RSA) was then assigned to each gene and a threshold of 0.01 was applied to identify genes differentially expressed across all donors in the same species (Konig et al., 2007). We also combined data from pre and post replicate measurements on one African green monkey host and one rhesus macaques host. All the original *log*_10_⁡*emPAI* scores were compared with those from replicates via linear regression yielding a standard deviation of 0.673. As a result, gene hits for cynomolgus and rhesus macaques were further filtered based on criteria of |*Avg*(*Log*_10_*FC*)| ≥0.673 and paired *t*-test *p* ≤ 0.05. A total of 115 unique genes were considered differentially expressed in at least one species and the hits were hierarchically clustered by FC of individual donor.

### Gene Function Enrichment Analysis

Enriched GO groups were identified for the 115 gene hits using Metascape, where GO groups with *p* ≤ 0.01 were retained (Zhou et al., 2019).

### Protein-Protein Network Analysis

Human PPI data used in this study were derived from Hynet^2^, Reactome^3^, BIND^4^, MINT^5^, HPRD^6^, and CORUM (Ruepp et al., 2008, 2010). A PPI network was first constructed by extracting direct interactions among the 115 genes, i.e., the MS hits, from the databases. Memišević et al. (2013) recently published two human gene lists and two mouse gene lists interacting with VFs using Y2H assays against whole human and whole murine proteome libraries. We performed permutation tests by randomly selecting 115 genes from human genome and computing the percentage of iterations ($P_{\text{permutation}}^{\text{L}}$) yielding more overlaps with the published gene list L or the extended gene list of L, i.e., genes in L and their PPI neighbors, out of 1,000 samplings. While $P_{\text{permutation}}^{L}$ for direct overlapping between the MS hits and the four lists ranged from 0.004 to 0.01, their extended gene lists were statistically significantly overlapped with all $P_{\text{permutation}}^{\text{L}}$ < 0.001.

### Preparation of Mouse Bone Marrow Derived Macrophages (mBMDMs)

Primary mBMDM were generated to investigate the impact of ezrin inhibitor, NSC668394 in modulating a panel of *B. mallei* mediated pro-inflammatory gene expression in physiologically relevant cell type. After euthanasia, 6-week-old female BALB/c mice were sprayed with 70% ethanol and the femurs were dissected using scissors, cutting through the tibia below the knee joints as well as through the pelvic bone close to the hip joint. Tibias were harvest by separating them from feet. Muscles connected to the bone were removed and the femurs and tibias were placed into a polypropylene tube containing sterile PBS on ice. In a tissue culture hood, the bones were placed in 70% ethanol for 1 min, washed in sterile PBS and then both epiphyses were removed. The bones were flushed with a syringe filled with RPMI 1,640 to extrude bone marrow into a 50 ml sterile polypropylene tube. After lysing the red blood cells, the cell suspension was cultured in RPMI 1,640 medium containing murine M-CSF. After 7 days, mBMDMs were harvested for treatment and infection as described below.

### *B. mallei* Infection *in vitro*

RAW264.7 macrophages were seeded at a density of 20,000 cells/well in 96-well imaging plates (Greiner μ clear). mBMDMs were seeded at density of 80,000 cells/well in regular tissue culture treated 96 well plates. Both cell types were then incubated at 37°C and 5% CO_2_. The next day, cells were pre-treated with the ezrin inhibitor NSC668394 at 20, 10, 5, and 2.5μM or equivalent amount of DMSO by volume. After 2 h, both macrophage types were infected with *B. mallei* (FMH23344) at a multiplicity of infection (MOI) of 1. After incubation for 2 h at 37°C and 5% CO_2_, extracellular bacteria were killed by addition of the antibiotic kanamycin (500 μg/ml). After incubation for 16 h at 37°C and 5% CO_2_, the infected RAW264.7 cells were fixed with 10% formalin for 24 h for subsequent immunofluorescence staining. The mBMDMs at 5 h post infection were processed for RNA isolation and Real-Time PCR studies.

### RNA Isolation and Real-Time PCR Analyses

BMDMs were lysed in TRIzol Reagent and total RNA was isolated according to the manufacturer’s protocol (Thermo Fisher Scientific, CA). Total RNA was reverse transcribed with iScript Reverse Transcription Supermix (Bio-rad, CA). cDNA samples were amplified using SYBR Green on the QuantStudio 12K Flex Real-Time PCR System (Thermo Fisher Scientific, CA). Briefly, the reaction conditions consisted of 2 μl of diluted cDNA with a final concentration of 150 nM primers in a 10 μl of reaction mix. Each cycle consisted of denaturation at 95°C for 15 s, annealing at 58.5°C for 5 s and extension at 72°C for 10 s. The specific primer sequences used for the real-time PCR are listed in Supplementary Table 2. TATA-binding protein (TBP) mRNA was used as an endogenous control to normalize each sample.

### Immunofluorescence Staining

Formalin fixed plates were washed with Dulbecco’s Phosphate buffered saline (DPBS), the cells were permeabilized for 15 min with DPBS containing 1% Triton X-100, washed and then blocked for 1 h with blocking buffer (Cellomics 1860291). To detect bacteria, infected cells were incubated for 1 h with goat anti-*Burkolderia mallei* polyclonal antibody (AB-G-BURK-M, BEI Resources) at 1:1,000 dilution. After subsequent washing and incubation for 1 h with the anti-goat Dylight 488 secondary antibody (1:500 dilution in blocking buffer), the cells were stained with Hoechst nuclear dye (Thermo Fisher Scientific, CA H3570, 1 mg/ml in PBS) and CellMask Deep Red dye (Thermo Fisher Scientific, CA H32721, 5 mg/ml in PBS) for host cell cytoplasmic staining.

## Results

### NHPs Exposed to Aerosolized *B. mallei* Exhibit Varying Clinical Signs of Disease Pathogenesis

The subjects consisted of six rhesus macaque, six African green monkeys and six cynomolgus macaques (Figure 1). Following exposure to *B. mallei*, the different NHP species showed different clinical signs. African green monkeys were by far very sensitive to *B. mallei*. Five of six animals developed lethal disease (1 animal on day 5 post-exposure and 4 animals between 11 and 14 days post-exposure); the surviving animal had an abscess and was bacteremic at study endpoint (45 days post-exposure to *B. mallei*). Cynomolgus and rhesus macaques were resistant to *B. mallei* and survived the challenge. All animals became febrile after exposure to *B. mallei* but most of the cynomolgus macaques exhibited the shortest duration of fever. In the African green monkeys and some of the rhesus macaques, body temperature remained elevated above baseline for the duration of the study.

African green monkeys exhibited leukocytosis with neutrophilia and monocytosis beginning 2–3 days post- infection, along with azotemia and elevation of the hepatic enzymes alanine aminotransferase (ALT) and aspartate aminotransferase (AST). Cynomolgus macaques had variable elevations of leukocytes during the study. Two of six cynomolgus macaques developed an apparent anemia of inflammatory disease. Leukocyte elevations in rhesus macaques were the most variable during the protocol. Four of six developed an apparent anemia of inflammatory disease.

Gross pathology and histopathology findings in the African green monkeys were consistent with reports of acute glanders in humans (data not shown): respiratory infection leading to bacteremia with pustule development in multiple organs and death within 2 weeks. It is important to note that little clinical data on acute human disease exist. Although there was pyogranulomatous or resolving inflammation with fibrosis in the lung and draining lymph nodes in five rhesus macaques, the lesions in other organs were limited, which is not necessarily consistent with acute glanders in humans. Cynomolgus macaques had few lesions in the lung and draining nodes and limited lesions in other systems. These observations are also not consistent with acute glanders in humans.

### Compilation and Conversion of Datasets Into Human Genes

To uncover the mechanisms of cellular responses to *B. mallei* infection, it is critical to study the protein expression profile from *B. mallei* infected primary cells using LC-MS/MS approaches. Profiling the abundance of the protein in primary cells provides a “snapshot” of the complex immune networks that operate throughput the system. The resulting mass spectra were interpreted automatically by querying MASCOT algorithm against NHP database (Perkins et al., 1999). In order to take advantage of the various bioinformatics knowledge databases and carry out functional and network analyses on proteomic dataset, we first mapped 5,093 protein sequence identification numbers (GI numbers) into genes. NCBI database search was performed to convert protein identifiers to gene identifiers, followed by homology search on non-human genes. Proteins without corresponding human genes were BLAST (Basic Local Alignment Search Tool) against all human proteins (Altschul et al., 1990). A threshold of 0.001 was applied to expectation value to retain human protein homologs. If there were multiple homologs for a protein, human gene(s) or homolog(s) the lowest expectation value was used. Combining database search and BLAST search, 5,036 protein sequences were mapped to 3,288 human genes (Supplementary Figure 1). Quantification of protein expressions were derived by the emPAI of the corresponding protein or the sum of emPAI scores if one gene was mapped to multiple proteins (Shinoda et al., 2010).

### Identification of Differentially Expressed Proteins in NHPs Upon *B. mallei* Infection

**FIGURE 2:**
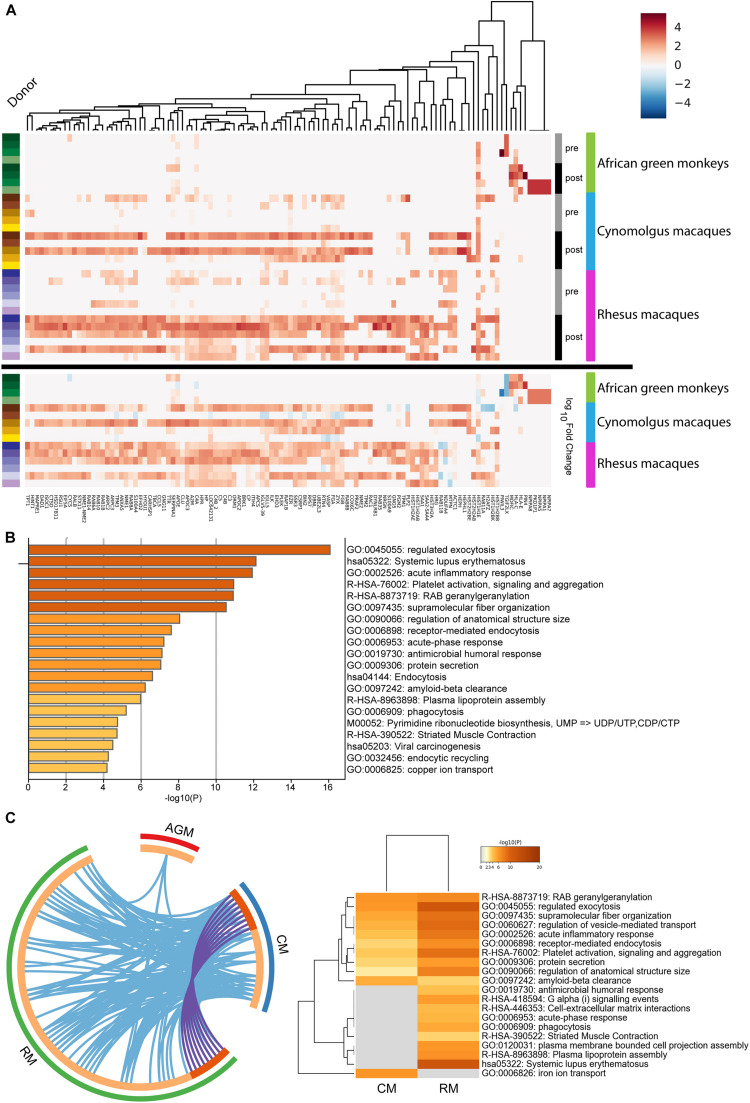
Hierarchical clustering of 115 proteins whose expressions were altered in *B. mallei* infected rhesus macaques, African green monkeys and cynomolgus macaques. **(A)** The expression pattern of 115 proteins was hierarchical clustered. A continuum of blue (low expression) to red (high expression) depicts expression levels of the protein. The heat maps under the “Log_1__0_Fold Change” bar depict the expression fold changes of proteins in logarithmic scale by normalizing *B. mallei* infected to uninfected samples. The heatmap under the “Pre” and “Post” bars represent expressions of PBMC derived proteins collected before and after *B. mallei* infection, respectively. Samples derived from individual donors were color coded (left panel). **(B)** Statistically significant overrepresentation of functional classes and protein families were presented. **(C)** The inner circle represents protein lists, where hits are arranged along the arc. Proteins that hit multiple lists are colored in dark orange, and proteins unique to a list are shown in light orange. Overlap of upregulated proteins between rhesus (RM) and cynomolgus macaques (CM) is represented as purple lines. Overlap of GO terms between rhesus macaques, African green monkeys (AGM) and cynomolgus macaques is represented as blue lines. All statistically enriched GO terms were first identified, accumulative hypergeometric *p*-values and enrichment factors were calculated and used for filtering. Remaining significant terms were then hierarchically clustered into a tree based on Kappa-statistical similarities among their gene memberships. 0.3 kappa score was applied as the threshold to cast the tree into term clusters.

**FIGURE 3:**
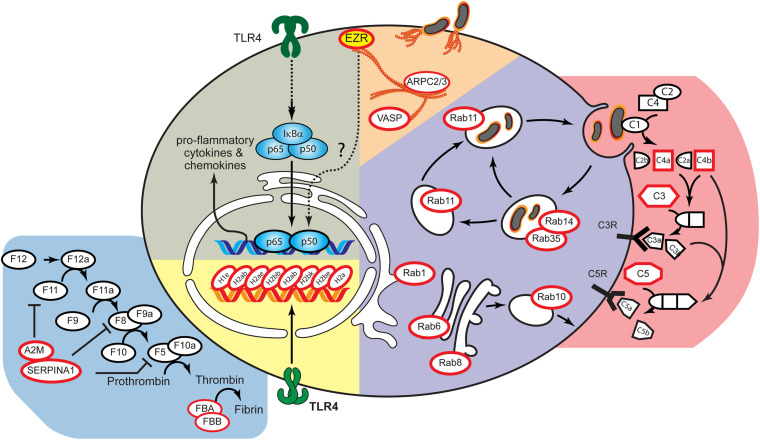
Integrated model of host factors whose expressions were altered after *B. mallei* infection. Using the *B. mallei* infection lifecycle as a guide (Galyov et al., 2010), the candidate proteins were placed at the position most likely to be relevant to the *B. mallei* infection using a database of annotations from Gene Ontology and Kyoto Encyclopedia of Genes and Genomes (KEGG). Red circle represent proteins whose expressions were altered after *B. mallei* infection. The yellow solid circles were validated host factors whose functions are involved in pro-inflammatory responses upon *B. mallei* infection.

To identify differentially expressed proteins before and after *B. mallei* infections, the 3,288 genes were subjected to statistical analysis (see Supplementary Figure 1 and experimental procedures). A total of 115 unique proteins were considered differentially expressed in at least one species and the hits were hierarchically clustered by the magnitude of fold changes of individual donor (Figure 2A). Of the 115 unique proteins, 86, 25, and 13 proteins were up-regulated in rhesus macaques, cynomolgus macaques and African green monkeys, respectively (Supplementary Table 3). GO analysis was performed to identify overrepresented biological annotations or protein families. This analysis elucidated 191 statistically enriched biological terms. Among others, a collection of proteins involved in complement and coagulation cascade, chromatin assembly as well as members of nucleoside metabolic process were implicated as factors likely to be important for host responses to *B. mallei* infection (*p* < 0.001) (Figure 2B and Supplementary Table 4). Furthermore, the expression of nine proteins was upregulated in both Rhesus and Cynomolgus macaques (Figure 2C and Supplementary Table 5). GO analyses suggested that these 9 proteins may function in vesicle-mediated transport, intracellular signal transduction (*p* < 0.001) and cytoskeletal protein binding (*p* < 0.003) (Figure 2C). The function of these 9 proteins may implicate their roles in mediating the replication of intracellular *B. mallei* whose life cycle relies on exploiting host proteins to gain motility and cell-to-cell spread. Integrated model of HFs whose expressions were altered after *B. mallei* infection is shown in Figure 3.

### Generation of Predictive Protein-Protein Interaction Network Between Host Factors and Virulence Factors

**FIGURE 4:**
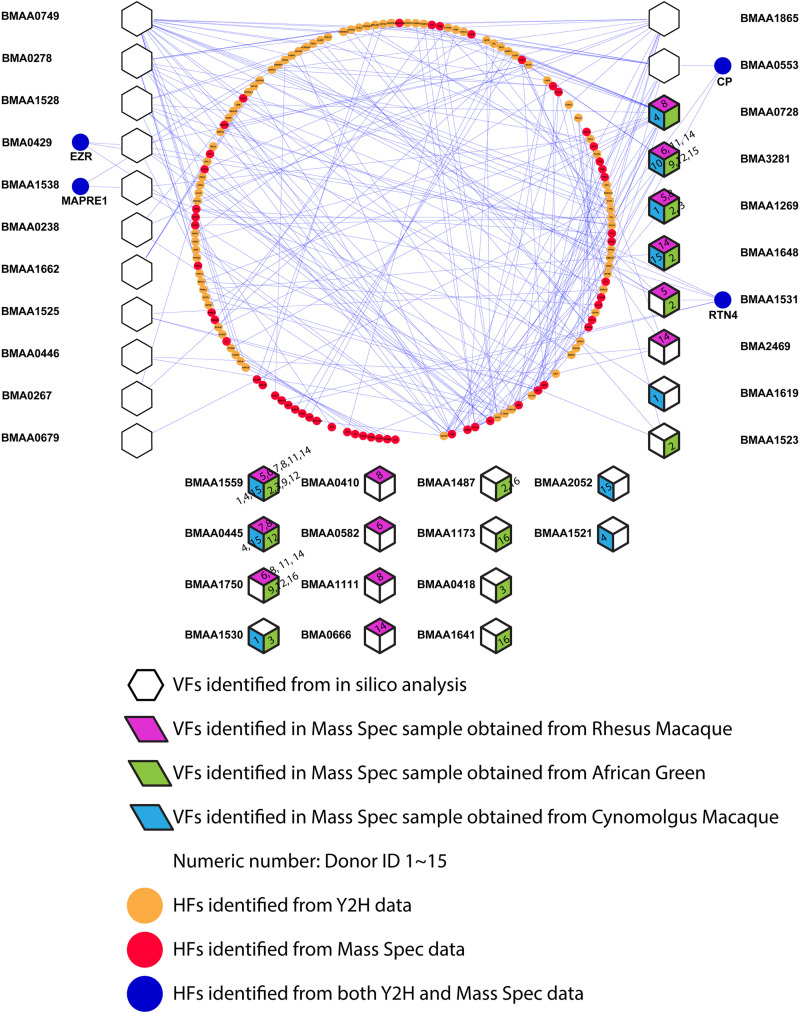
The network relationship between identified host factors and *B. mallei* virulence factor is depicted. Hexagons indicate *B. mallei* virulence factors identified by Memišević et al. (2013). Red, green and blue diamonds indicate the presence of *B. mallei* virulence factor in rhesus macaque, African green monkey and cynomolgus macaque, respectively. Orange and Red nodes depict host factors identified by Memišević et al. (2013) using Y2H method or LC-MS/MS approach, respectively. Blue nodes depict host factors identified by both Y2H and LC-MS/MS approach. The digits on the diamonds represent the identification number of the donor NHPs.

Memišević et al. (2013). reported the identification of 49 *B. mallei* VFs through a combination of computational and experimental approaches. Using the Y2H assay system, 26 of the *B. mallei* virulence factors and their interacting HFs were curated. To gain insights toward the relationship between *B. mallei* VFs versus 115 HFs identified in our LC-MS/MS study, a predictive PPI network based on the binary protein-protein interaction data obtained from the Y2H study was generated (Figure 4). The overall PPI network is composed of 21 *B. mallei* virulence factors that either directly or indirectly interact with 140 host proteins. Of these 140 host proteins, 54 and 82 host proteins were identified in either LC-MS/MS or Y2H analyses, respectively. Importantly, four host proteins were identified in both LC-MS/MS and Y2H analyses. These four proteins are ezrin, Microtubule-associated protein RP/EB family member 1 (MAPRE1), ceruloplasmin (CP) and Reticulon-4 (RTN4). The spectra from LC-MS/MS dataset were also queried against the *B. mallei* protein database. Twenty-two VFs were found to be present in at least one of the donors in one of the NHPs. Eight of the VFs were able to interact with the major PPI network whereas 14 additional VFs could not be mapped to the PPI network due to the lack of Y2H data. Interestingly, among these 22 VFs, 5 of them were found in all three NHP species (BMA3281, BMAA1269, BMAA1648, BMAA1559, BMAA0445), suggesting the importance of these factors in mediating host response as well as bacterial replication during the pathogenesis of *B. mallei* infection.

### Inhibiting Ezrin Reduced *B. mallei* Mediated Pro-inflammatory Gene Up-Regulation

**FIGURE 5:**
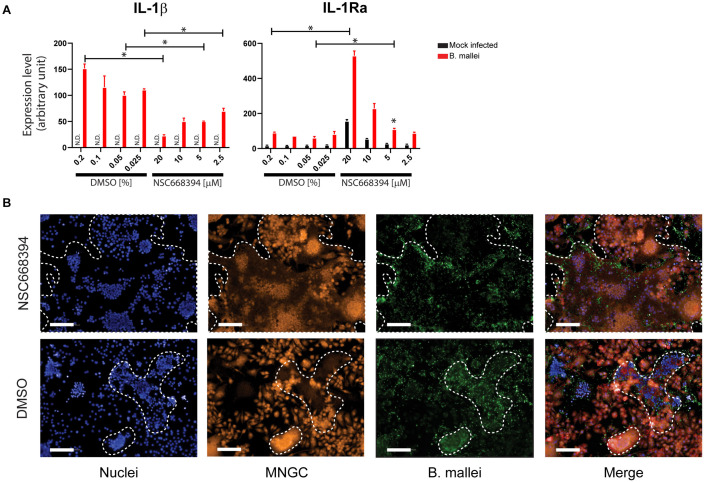
Ezrin modulates *B. mallei* mediated pro-inflammatory responses. **(A)** Two hours before *B. mallei* infection (MOI = 1), mBMDMs were pretreated with ezrin inhibitor at indicated concentrations. Total RNAs were extracted 5 h post infection and the expression level of indicated genes were quantified by real-time PCR. N.D. indicates not detected. “^∗^” denotes *p* < 0.05. **(B)** Two hours before *B. mallei* infection (MOI = 1), RAW264.7 macrophages were pretreated with ezrin inhibitor (10 μM, images shown). The cells were fixed 16 h post infection. Orange stain: cytoplasm; Green stain: *B. mallei*; Blue stain: nuclei. The MNGC formation is traced in white. (Data representing two biological replicates which include 6 analytical replicates).

rThrough the constructed predictive PPI network, we next investigated the involvement of Ezrin, a (HF) in mediating *B. mallei* induced inflammatory responses. Ezrin is a multifunctional protein that connects the actin cytoskeleton to the extracellular matrix through transmembrane proteins (Bulut et al., 2012). Furthermore, it was demonstrated that loss of ezrin in B cells results in dampened proinflammatory response to a sublethal dose of lipopolysaccharide *in vivo* (Pore et al., 2016). In our studies, ezrin expression was elevated upon *B. mallei* infection, and in the Y2H study, it was also shown to directly interact with BMA0429, a cytidylate kinase that is involved in pyrimidine metabolism (Memišević et al., 2013). BMA0429 is deemed essential to *B. mallei* because it is orthologous to STY0980, an essential gene of *Salmonella enterica* serovar Typhimurium (Figure 4; Langridge et al., 2009). We investigated the impact of the ezrin inhibitor, NSC668394, in modulating a panel of *B. mallei* mediated pro-inflammatory gene expressions in physiologically relevant primary mBMDMs. Real-time PCR analysis revealed NSC668394 specifically impinged on *B. mallei* mediated IL-1β and IL-1Ra expressions. mBMDMs pretreated with NSC668394 followed by *B. mallei* infection demonstrated reduced mRNA levels of IL-1β whereas IL-1Ra was potentiated in a dose dependent manner (Figure 5A). The expression levels of other inflammation related cytokines and chemokines such as TNF-α, CCL2, CCL3, CCL5, CCL7, CCL12, and CCL17 were unchanged (Supplementary Figure 2). Furthermore, RAW264.7 macrophages pretreated with NSC668394 showed more pronounced MNGC formation when compared to DMSO control (Figure 5B). Taken together, these data suggest that inhibiting ezrin by NSC668394 specifically reduced IL-1β and potentiated IL-1Ra mRNA upregulation, which correlated to elevated MNGC formation upon *B. mallei* infection.

## Discussion

To satisfy the regulatory requirements for the approval of countermeasures against high-consequence pathogens, the FDA instituted the Animal Rule, which permits efficacy studies in animal models in place of human clinical data when such studies are not feasible or ethical. Therefore, the development and detailed study of animal models for human bacterial infections are absolutely critical for understanding disease pathogenesis or evaluating therapeutic interventions, and vaccine modalities *in vivo*. Although small animals, particularly rodents, are a mainstay for biological and immunological research, numerous biological phenomena that occur in humans cannot be modeled in mice. While NHP species such as the African green monkey, the rhesus macaque, and the cynomolgus macaque have been successfully used as animal models that mimic human infections for biothreat agents such as *Yersinia pestis*, *Bacillus anthracis*, and *Coxiella burnetti*, there is few data on the clinical course of aerosolized *B. mallei* infections in NHP’s (Henning et al., 2012; Hammamieh et al., 2016; Gregory et al., 2019).

The induction of innate immunity, inflammatory cytokines and chemokines following *B. mallei* infection have been reported in *in vitro* and rodent models; however, a global characterization of host responses toward glanders at the molecular level has never been investigated systematically using a NHP model. By utilizing the LC-MS/MS approach, we identified host proteins with altered expression in PBMCs obtained from rhesus macaques, African green monkeys and cynomolgus macaques exposed to *B. mallei* infection. A statistically significant PPI network was constructed by integrating the dataset obtained from LC-MS/MS with the previously published *B. mallei* host-pathogen protein interaction database curated from Y2H approach (Memišević et al., 2013). We identified ezrin, a member of the ERM (Ezrin/Radixin/Moesin) proteins, whose expression was induced after *B. mallei* infection and it also interacts with multiple *B. mallei* VFs. The ezrin inhibitor specifically reduced *B. mallei* mediated IL-1β expression. Furthermore, murine macrophages pre-treated with the ezrin inhibitor showed prominent MNGC formation, a hallmark of *B. mallei* infection. This suggested that ezrin plays a role in modulating IL-1β expression which may ultimately impact the pathogenesis of *B. mallei*. Although the magnitude of MNGC formation and its correlation with *B. mallei* virulence are unknown, insight could be gained from the infection of *B. pseudomallei*, a bacterium that is closely related to *B. mallei* in the *Burkholderia* genus. Welkos et al. (2015) observed a reproducible correlation indicating that the lesser virulent strains *B. pseudomallei* (as determined by murine models of melioidosis) demonstrated significantly enhanced MNGC formation rate and improved bacterial survival in J774.A1 murine-derived macrophage-like cells. By employing a systems biology approach, we have revealed the topology of a protein interaction network, which reflects the underlying host-pathogen interactions of *B. mallei* infection in a NHP model.

### Ezrin Modulates IL-1β Signaling Axis Activity During *B. mallei* Infection

The underlying host molecular signaling pathways that govern *B. mallei* pathogenesis is not well characterized. However, insight could be gained from the infection of *B. pseudomallei* (Hatcher et al., 2015). Ceballos-Olvera et al. (2011) presented multiple lines of evidence suggesting that IL-1β is deleterious in the mouse lung infection model. For example, IL-1β deficient mice and wild type mice that were exogenously administered with either IFN-γ or IL-1Ra are more resistant to *B. pseudomallei* infection whereas mice that exogenously received IL-1β are prone to infection. The deleterious role of IL-1β is due to several reasons including excessive recruitment of neutrophils, which may support intracellular growth of *B. pseudomallei*, increase tissue damage, and inhibition of IFN-γ production. On the contrary, IL-1Ra terminates the pro-inflammatory signal cascade by competing with IL-1β for binding to the cognate receptor. Here, we report that the ezrin inhibitor, NSC668394, not only reduced *B. mallei* mediated IL-1β mRNA expression but also potentiated IL-1Ra mRNA expression. The dual activities of NSC668394 may ultimately reduce IL-1β mediated pro-inflammatory responses during *B. mallei* infection and promote the survival of the host. Aside from this observation, ezrin is historically recognized as a mediator of actin polymerization. Given that actin polymerization is a critical process in *B. mallei* life cycle, the impact of ezrin in modulating *B. mallei* mediated actin polymerization requires further investigation.

### Indication of Rab Family of GTPases in Modulating Host Responses to *B. mallei* Infections

Multiple Rab family of GTPases were upregulated in PBMCs after *B. mallei* infection (Figure 3). The Rab family of GTPases orchestrates membrane transport signaling to combat bacterial pathogen invasion in mammalian host. Rab proteins help create vacuoles which envelope bacteria and eventually lead to bacterial cell lysis. However, highly pathogenic bacteria have evolved myriad mechanisms to perturb membrane transport and replicate within the vacuoles, relying heavily on the secretion of proteins that perturb the more than 60 host Rab GTPases which control membrane transport (Brumell and Scidmore, 2007; Sherwood and Roy, 2013). A dramatic example of pathogen induced Rab perturbation includes *Vibrio cholera* which directly modulates Rab11 to damage the epithelial integrity of the mammalian gut leading to the loss of 10–20 L fluid per day in infected humans (Guichard et al., 2014). As part of the host defense mechanism to infection, Rab proteins help control the killing of bacteria following phagocytosis (Mottola, 2014). However, bacteria can interfere with the Rab protein signaling network to promote infection in the host. Notable examples include bacterial effectors which block the well-established Rab5 positive early phagosome to Rab7 enriched late phagosome transition. By either blocking the recruitment of Rab5 or the transition from Rab5 positive to Rab7 positive phagosomes, pathogenic bacteria can rapidly establish a host vacuole for intracellular replication which never gets fused with the lysosome (Mottola, 2014). *Burkholderia cenocepacia* blocks late phagosome fusion to the lysomome via inactivation of Rab7 (Huynh et al., 2010). Other targets abrogating transport of the vacuole to the lysosome include Rab32, a host protein which has its activity blocked by *Salmonella enterica* serovar Typhi (Spano et al., 2016). The entire family of GTPases are important targets of bacterial effectors (Ham et al., 2011); however, the Rab GTPase are especially enriched targets for modulating the host environment to facilitate bacterial replication (Ham et al., 2011; Stein et al., 2012).

### NHP Model Demonstrated Commonalities Between Host Responses After *B. mallei* or Monkey Pox Infections

Previously, proteomic analysis of lung fluids collected from NHPs infected with Monkeypox virus or Vaccinia virus revealed that 76% of the proteins involved in the coagulation and complement cascades were up-regulated in both these infection models (Brown et al., 2010). Our proteomic interaction analysis also revealed upregulation of components in the coagulation and complement cascades following *B. mallei* infection. These proteins include C4a, C4b, C3, C5, A2M, SERPINA1, FBA and FBB (Figure 3). Our results, coupled with previous proteomic studies, confirm that the activation of the complement cascade is a common innate immune response against invading pathogens.

In this study, we report the expression levels of 86, 25, and 13 proteins that are altered in rhesus macaques, cynomolgus macaques and African green monkeys, respectively, following Bm infection. Importantly, the expression of nine proteins was induced in both rhesus and cynomolgus macaques. Integrating the two orthogonal proteomic datasets enabled the construction of a statistically significant predictive PPI host pathogen interaction network. The topology of the PPI network revealed 58 proteins interacted with one of the 21 *B. mallei* VFs directly or indirectly. GO analysis revealed proteins involved in complement and coagulation cascade, chromatin assembly as well as members of nucleoside metabolic process that may play an important role in host responses to *B. mallei* infection. The network study revealed that BMA0429, a cytidylate kinase that is essential to *B. mallei*, directly interacts with host protein ezrin, whose expression is upregulated in the PBMCs of *B. mallei* infected rhesus macaques. The ezrin inhibitor specifically impinged on *B. mallei* mediated IL-1β and IL-1Ra expressions levels, thus suggesting that the host protein ezrin could serve as a potential target for therapeutic discovery. Furthermore, the predictive PPI network derived from NHPs provided a comprehensive view between cellular host proteins and *B*. *mallei* VFs.

## Data Availability Statement

The datasets presented in this study can be found in online repositories. The names of the repository/repositories and accession number(s) can be found in the article/Supplementary Material.

## Ethics Statement

Animal research at the United States Army Medical Research Institute of Infectious Diseases (USAMRIID) was conducted under an animal use protocol approved by the USAMRIID Institutional Animal Care and Use Committee (IACUC) in compliance with the Animal Welfare Act, PHS Policy, and other Federal statutes and regulations relating to animals and experiments involving animals. The facility is accredited by the Association for Assessment and Accreditation of Laboratory Animal Care International (AAALAC) and adheres to principles stated in the Guide for the Care and Use of Laboratory Animals (National Research Council, 2011).

## Author Contributions

C-YC, RP, and PW conceived and designed the experiments. C-YC, MW, DL, TK, RR-A, BE, ST, TC, DW, LC, and CC performed the experiments. YZn and YZu analyzed the data. C-YC, RM, and RP wrote the manuscript. All authors contributed to the article and approved the submitted version.

## Disclaimer

Opinions, interpretations, conclusions, and recommendations are those of the authors and are not necessarily endorsed by the U. S. Army.

## Conflict of Interest

The authors declare that the research was conducted in the absence of any commercial or financial relationships that could be construed as a potential conflict of interest.

## Footnotes

1 http://www.ncbi.nlm.nih.gov/

2 https://ostr.ccr.cancer.gov/bioinformatics/software/pathwaystudio/

3 http://reactome.org

4 http://baderlab.org/BINDTranslation

5 https://pubmed.ncbi.nlm.nih.gov/31945268/

6 http://www.hprd.org

## Supplementary Material

Supplementary Figure 1 Schematic of compiling and converting datasets into human genes. 5,093 GIs were subjected for NCBI search. 408 GIs corresponded to human genes. 2,110 GIs corresponded to non-human gene and 2,575 GIs had no matches. 2,110 GIs were further subjected for HomoloGene search. 1,483 GIs corresponded to human gene and 627 GIs had no matches. A total of 2,575 unmatched GIS after NCBI search plus 627 unmatched GIs after HomoloGene search were BLAST; 3,145 GIs were identified to correspond to human genes. After filtering out redundant genes, 3,288 unique genes were retained for downstream analyses.

Supplementary Figure 2 *B. mallei* mediated pro-inflammatory gene expressions. Two hours before *B. mallei* infection, mBMDM were pretreated with ezrin inhibitor at various concentrations. Total RNAs were extracted 5 h post infection and the expression level of indicated genes were quantified by real-time PCR.

Supplementary Table 1 The time of PMBCs that were collected post infections.

Supplementary Table 2 The primer sequences used for the real-time PCR in this study.

Supplementary Table 3 Hierarchical clustering of the proteomic data. Tab “Data” is the underlying data for Figure 2. African Green monkey (AGM), cynomolgus macaques (CM) and rhesus macaques (RM).

Supplementary Table 4 Gene ontology (GO) analyses of 86 host factors that are differentially expressed in *B. mallei* infected rhesus macaques. Tab “Data” is the underlying data for Figure 2B.

Supplementary Table 5 The underlying data of the Circo plot and share GO terms for Figure 2C.

Supplementary Table 6 The mass spectrometry proteomics data has been deposited to the ProteomeXchange Consortium via the PRIDE partner repository. The dataset identifiers are listed in the Table.
