# Supplementary figures and images for: Proteomic Analysis of Non-human Primate Peripheral Blood Mononuclear Cells During Burkholderia mallei Infection Reveals a Role of Ezrin in Glanders Pathogenesis
Source: Front Microbiol. 2021 Apr 22;12:625211. doi: 10.3389/fmicb.2021.625211 (PMC8101288; doi:10.3389/fmicb.2021.625211)

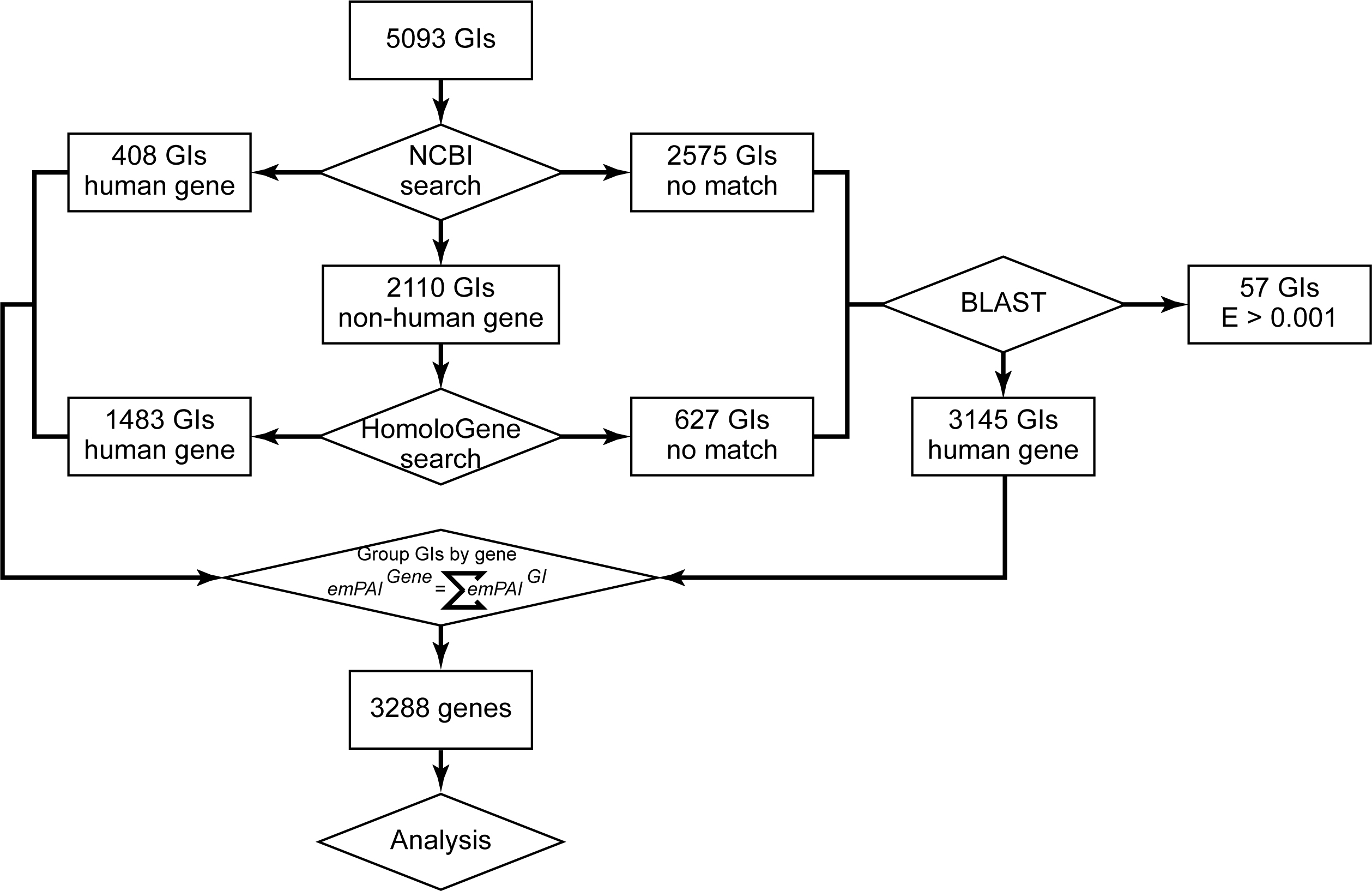

Supplement: Supplementary Figure 1 — Schematic of compiling and converting datasets into human genes. 5,093 GIs were subjected for NCBI search. 408 GIs corresponded to human genes. 2,110 GIs corresponded to non-human gene and 2,575 GIs had no matches. 2,110 GIs were further subjected for HomoloGene search. 1,483 GIs corresponded to human gene and 627 GIs had no matches. A total of 2,575 unmatched GIS after NCBI search plus 627 unmatched GIs after HomoloGene search were BLAST; 3,145 GIs were identified to correspond to human genes. After filtering out redundant genes, 3,288 unique genes were retained for downstream analyses. [file Image_1.JPEG]

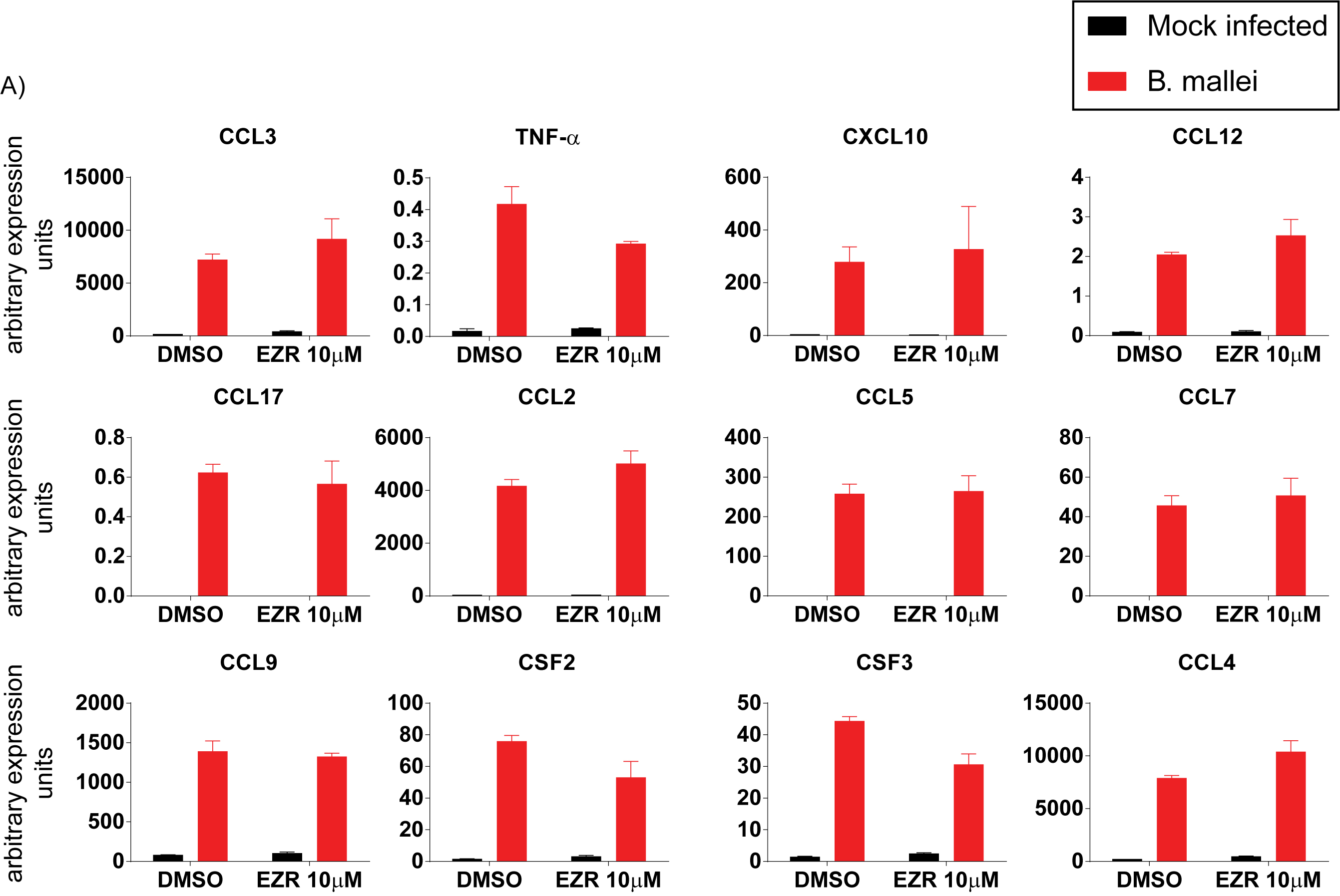

Supplement: Supplementary Figure 2 — B. mallei mediated pro-inflammatory gene expressions. Two hours before B. mallei infection, mBMDM were pretreated with ezrin inhibitor at various concentrations. Total RNAs were extracted 5 h post infection and the expression level of indicated genes were quantified by real-time PCR. [file Image_2.JPEG]
